# The Effects of Cropping Regimes on Fungal and Bacterial Communities of Wheat and Faba Bean in a Greenhouse Pot Experiment Differ between Plant Species and Compartment

**DOI:** 10.3389/fmicb.2017.00902

**Published:** 2017-05-29

**Authors:** Sandra Granzow, Kristin Kaiser, Bernd Wemheuer, Birgit Pfeiffer, Rolf Daniel, Stefan Vidal, Franziska Wemheuer

**Affiliations:** ^1^Section of Agricultural Entomology, Department of Crop Sciences, University of GöttingenGöttingen, Germany; ^2^Genomic and Applied Microbiology and Göttingen Genomics Laboratory, Institute of Microbiology and Genetics, University of GöttingenGöttingen, Germany; ^3^Plant Nutrition and Crop Physiology, Department of Crop Sciences, University of GöttingenGöttingen, Germany

**Keywords:** microbial diversity, multiple cropping vs. monoculture, microbial interactions, indicator species, co-occurrence networks

## Abstract

Many bacteria and fungi in the plant rhizosphere and endosphere are beneficial to plant nutrient acquisition, health, and growth. Although playing essential roles in ecosystem functioning, our knowledge about the effects of multiple cropping regimes on the plant microbiome and their interactions is still limited. Here, we designed a pot experiment simulating different cropping regimes. For this purpose, wheat and faba bean plants were grown under controlled greenhouse conditions in monocultures and in two intercropping regimes: row and mixed intercropping. Bacterial and fungal communities in bulk and rhizosphere soils as well as in the roots and aerial plant parts were analyzed using large-scale metabarcoding. We detected differences in microbial richness and diversity between the cropping regimes. Generally, observed effects were attributed to differences between mixed and row intercropping or mixed intercropping and monoculture. Bacterial and fungal diversity were significantly higher in bulk soil samples of wheat and faba bean grown in mixed compared to row intercropping. Moreover, microbial communities varied between crop species and plant compartments resulting in different responses of these communities toward cropping regimes. Leaf endophytes were not affected by cropping regime but bacterial and fungal community structures in bulk and rhizosphere soil as well as fungal community structures in roots. We further recorded highly complex changes in microbial interactions. The number of negative inter-domain correlations between fungi and bacteria decreased in bulk and rhizosphere soil in intercropping regimes compared to monocultures due to beneficial effects. In addition, we observed plant species-dependent differences indicating that intra- and interspecific competition between plants had different effects on the plant species and thus on their associated microbial communities. To our knowledge, this is the first study investigating microbial communities in different plant compartments with respect to multiple cropping regimes using large-scale metabarcoding. Although a simple design simulating different cropping regimes was used, obtained results contribute to the understanding how cropping regimes affect bacterial and fungal communities and their interactions in different plant compartments. Nonetheless, we need field experiments to properly quantify observed effects in natural ecosystems.

## Introduction

In the last decades, multiple or mixed cropping systems have received more attention due to their potential for a sustainable intensification of agriculture (Vandermeer, 1992). They provide beneficial ecological and economical services such as reduced plant pathogen damage (Winter et al., 2014). In addition, multiple cropping systems enhance plant productivity by improving the exploitation of available resources (Zhang and Li, 2003; Hauggaard-Nielsen and Jensen, 2005). Previously, it was suggested that (positive) interspecific interactions in the rhizosphere (Li et al., 1999; Zhang and Li, 2003) or changes in microbial communities and chemical soil properties may also be responsible for increased yields (Song et al., 2007b).

Bacteria and fungi play essential roles in biogeochemical cycling of matter and thus ecosystem functioning (Ellouze et al., 2014; van der Heijden and Hartmann, 2016). Many of them are beneficial to plant nutrient acquisition, health, and growth in the plant's rhizosphere and endosphere (Lugtenberg and Kamilova, 2009; Philippot et al., 2013). These microorganisms may also alleviate abiotic stress conditions of their host plants (Malinowski and Belesky, 2000; de Zelicourt et al., 2013). In addition, they can enhance the resistance of their host plant against biotic stressors such as herbivores or plant pathogens (Siddiqui and Shaukat, 2003; Vidal and Jaber, 2015).

Previous studies have addressed the role of cropping systems on microbial communities in endosphere and rhizosphere soil (e.g., Song et al., 2007a; Zhang et al., 2011, 2015). Song et al. (2007a) analyzed ammonia-oxidizing bacteria in the rhizosphere of intercropped wheat, maize, and faba bean using denaturing gradient gel electrophoresis (DGGE) and reported differences in the bacterial community structure when comparing intercropping systems and monocultures. However, most research focused on microorganisms in the rhizosphere and/ or on ammonia-oxidizing bacteria only (Song et al., 2007a; Zhang et al., 2015; Li et al., 2016). So far, entire bacterial and fungal communities and their interactions in different plant compartments of two important crop species under different cropping regimes have not been studied simultaneously using large-scale metabarcoding.

Hence, we investigated the influence of cropping systems on plant-associated fungal and bacterial communities using metabarcoding. The current study is embedded in the IMPAC^3^ project (“Novel genotypes for mixed cropping allow for improved sustainable land use across arable land, grassland and woodland”). To assess structural changes of the studied microbial communities with respect to cropping system or plant species, a greenhouse pot experiment was designed simulating different cropping regimes under controlled conditions. For that purpose, the two crop species winter wheat (*Triticum aestivum* L.) and winter faba bean (*Vicia faba* L.) were grown in monoculture and in two different intercropping regimes, i.e., row and mixed intercropping. We used row and mixed intercropping as previous studies have shown that various intercropping regimes influenced facilitative and competitive interactions between intercropped plant species in a different manner due to differences in root systems (Li et al., 1999; Mariotti et al., 2009), which might affect the plant microbiome as well. Bacterial and fungal communities in bulk and rhizosphere soil as well as in aerial plant parts and root endosphere were examined using Illumina (MiSeq) sequencing targeting the bacterial 16S rRNA gene and the fungal internal transcribed spacer (ITS) region, respectively. Our major aims were as follows: (i) to assess the effect of different cropping regimes on microbial diversity and community structures, (ii) to examine whether this effect differs between plant species and plant compartments as microbial communities most properly exhibit plant species-specific and plant compartment-specific structures, and (iii) to determine whether intercropping regimes decrease the number of negative interactions within the microbial community. Obtained results will further deepen our understanding of how cropping regimes influence the plant microbiome.

## Materials and methods

### Experimental design

To examine the influence of cropping systems on the entire fungal and bacterial community in soil and endosphere, we developed an experimental system to simulate monoculture and two intercropping settings in agriculture. For this purpose, the two crop species winter faba bean (genotype: Hiverna) and winter wheat (genotype: Hybery) were planted in monoculture or as mixture in polypropylene containers (Semadeni, Eurobehälter, LogiLine® SGL Boden, 600 × 400 × 212 mm) in summer 2015. Each container contained 25% sand and 75% non-sterile commercial plant substrate (Fruhstorfer Erde Typ T25; N: 200–300 mg L^−1^, P_2_O_5_: 200–300 mg L^−1^; Hawita Gruppe GmbH Vechta, Germany). This commercial plant substrate is a peaty soil with a pH (CaCl_2_) of 5.5–6.5. We used this homogenous growth substrate for the experiment to maintain constant abiotic soil conditions across the different cropping regimes. The soil was not autoclaved or steamed. In addition, the seeds were not surface-sterilized prior to planting.

**Figure 1:**
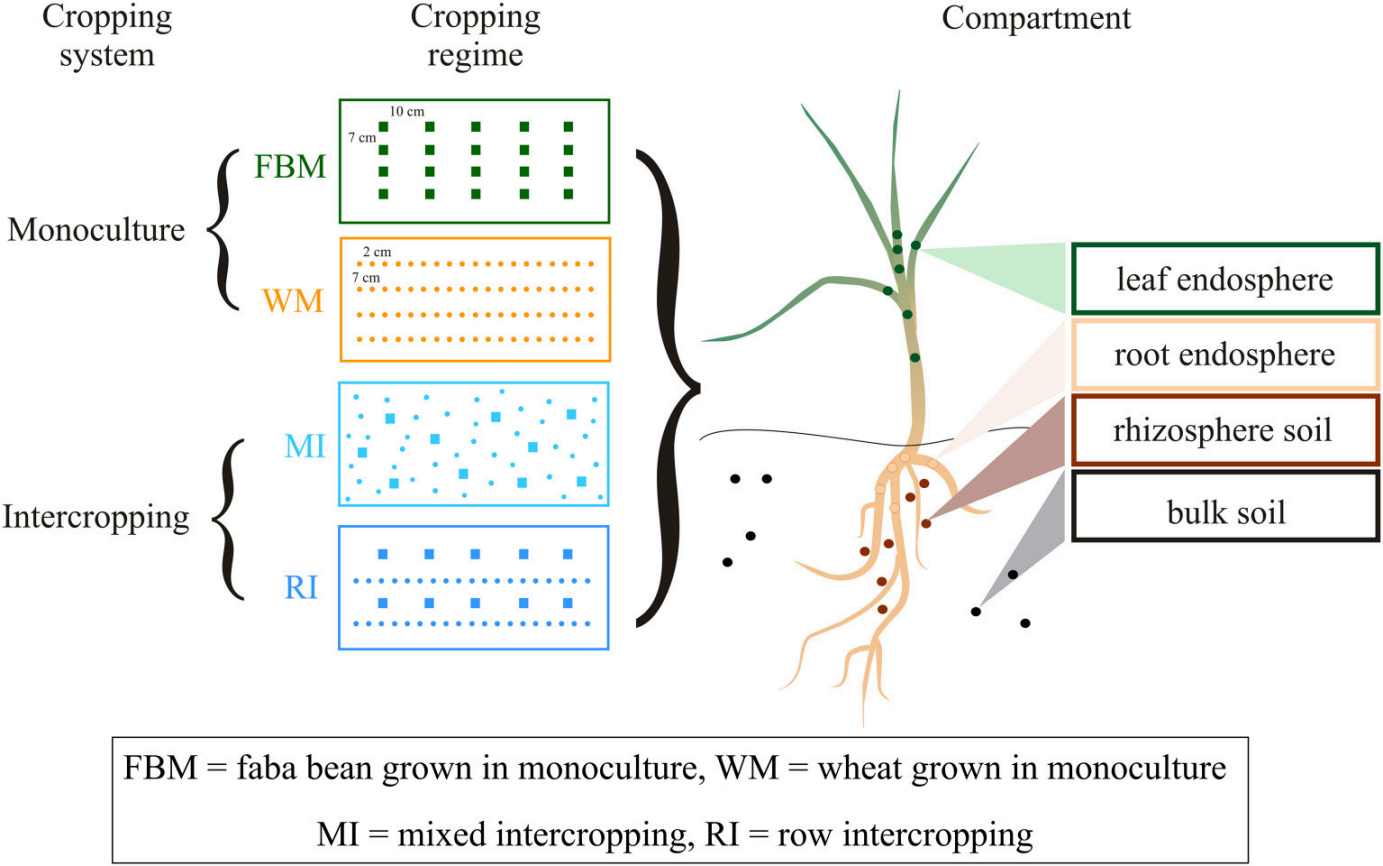
**Experimental design of the study**. Faba bean and wheat were grown in two cropping systems (monoculture vs. intercropping) resulting in four cropping regimes. Fungal and bacterial communities in four compartments were studied: rhizosphere and bulk soil as well as root and aerial (here regarded as leaf) endosphere.

For monocultures, 20 faba bean (FBM) or 80 wheat (WM) plants per container were sawn in rows (Figure 1). In multiple cropping systems, 40 wheat and 10 faba bean plants per container were grown either in distinct rows (row intercropping; RI) or without any distinct row arrangement (mixed intercropping; MI) as defined by Andrews and Kassam (1976). We distinguished between cropping systems (monoculture vs. multiple cropping systems) and cropping regimes (WM, FBM, MI, and RI). Each cropping regime was replicated five times in a randomized block design. All plants were cultured under normal diel light cycles in a semi-closed greenhouse and irrigated daily for a growing period of 4 weeks. We chose controlled greenhouse conditions to reduce the entrance of pests and the variation from other environmental factors. No fertilizer treatments were applied to increase nutrient-limitation as well as intra- and inter-species interactions between the plants. Fungal and bacterial communities in four compartments of healthy plants were studied: the rhizosphere and bulk soil as well as the root and aerial (here regarded as leaf) endosphere (Figure 1).

### Soil sampling and edaphic parameters

After a growing period of 4 weeks, we sampled the rhizosphere soil, defined as soil tightly adhering to the roots, and the bulk soil, defined as root-free soil around the crops. In the two intercropping treatments, bulk soil samples of the two crop species were pooled for each container resulting in 20 bulk soil samples (Table 1). The roots were gently shaken to remove the non-rhizosphere soil. Rhizosphere soil, tightly attached to root surface, was collected by carefully brushing the roots. Ten subsamples were collected for each container, and obtained rhizosphere soil samples were thoroughly mixed in order to obtain one single sample. A total of 30 rhizosphere soil samples was collected. All soil samples were frozen and stored at −20°C.

**Table 1:** **Sampling numbers**.

	**Bulk soil**	**Rhizosphere**	**Roots**	**Leaves**	**Plants/treatment**
**COMPARTMENTS**					
WM	5 (5/5)	5 (5/5)	5 (5/4)	5 (5/5)	50
FBM	5 (5/5)	5 (5/5)	5 (4/4)	5 (3/2)	25
W_MI	5^*^ (5/5)	5 (4/4)	5 (5/5)	5 (5/5)	50
FB_MI	5^*^ (5/5)	5 (5/5)	5 (5/5)	5 (2/3)	25
W_RI	5^*^ (5/5)	5 (5/5)	5 (5/5)	5 (5/5)	50
FB_RI	5^*^ (5/5)	5 (5/5)	5 (5/3)	5 (5/5)	25
Total	20	30	30	30	150 (W), 75 (FB)

*In total, 150 wheat and 75 faba bean plants were collected*.

* *Bulk soil samples of both plants in the intercropping regimes were pooled prior analysis. Numbers in brackets refer to the number of samples left after removal of samples with too low sequencing numbers. The first number refers to bacteria, the second to fungi. W, wheat; FB, faba bean; FBM, faba bean in monoculture; WM, wheat in monoculture; FB_MI, faba bean samples from mixed intercropping; FB_RI, faba bean samples from row intercropping; W_MI, wheat samples from mixed intercropping; W_RI, wheat samples from row intercropping; MI, samples from mixed intercropping; RI, samples from row intercropping*.

For determination of soil properties, subsamples were dried at 60°C for 2 days and sieved to <2 mm. Soil organic carbon (C) and total nitrogen (N) concentrations from all dried subsamples were determined using a LECO TruSpec CN analyser (Leco Corp., St. Joseph, MI). The gravimetric soil water content (%) of all soil samples was calculated from oven-dried subsamples. Soil pH-values were measured as follows: 2 g soil of each container was mixed with 5 mL PCR grade water. After incubation for 24 h, pH_Water_ was measured in the supernatant with a glass electrode. Subsequently, 0.37 g KCl was added and pH_KCl_ was measured. Details on edaphic parameters are provided in Table S1.

### Sampling and plant growth characteristics

**Figure 2:**
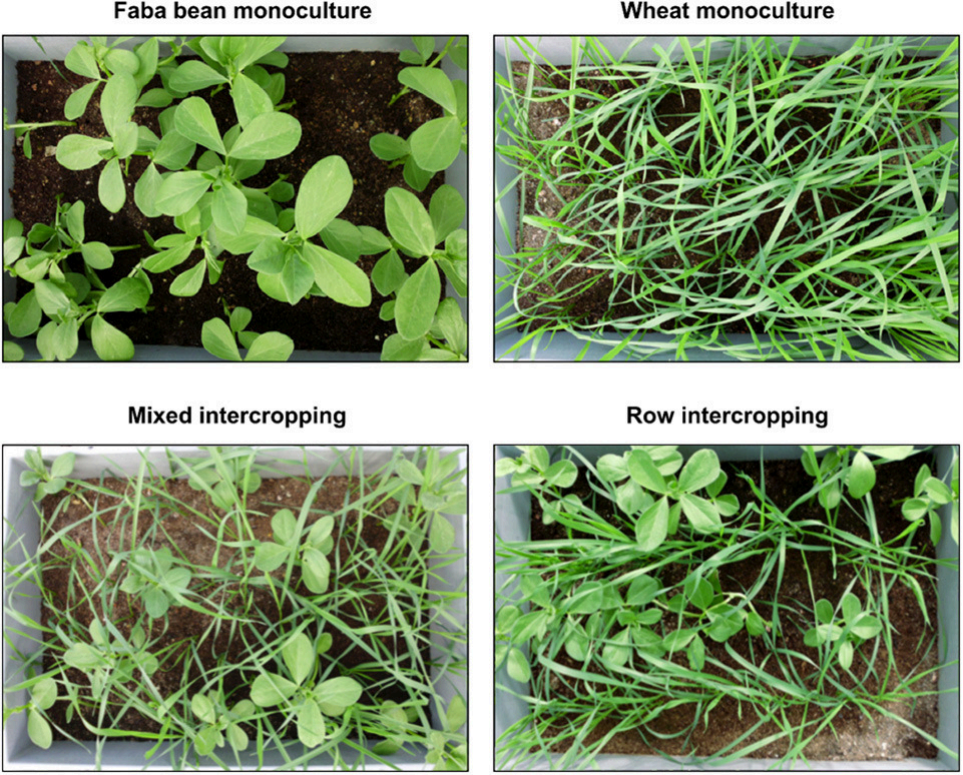
**Plants of the four cropping regimes**. Photos were taken 3 days before harvesting.

Above- as well as belowground plant material of the two crop species were harvested separately for each container at a BBCH of 14–16 (wheat) or 15–18 (faba bean). The BBCH-scale describes the developmental stages of Mono- and Dicotyledonous weed species (Hess et al., 1997). Aboveground (shoots, leaves) and root biomass for each crop species and each container were measured (Table S2). In addition, the heights of 10 faba bean and 20 wheat plants in intercropping regimes and 20 plants of monocultured faba bean and wheat plants were measured. For determination of water content in aerial plant parts, 10 wheat and five faba bean plants without roots per container were weighted and subsequently oven-dried at 60°C for 48 h and re-weighted (Table S2). Ten wheat and five bean plants, which did not show any obvious disease symptoms (Figure 2), were randomly selected from each container for further molecular analysis. In total, 75 faba bean and 150 wheat plants were collected. Plant material derived from the same container and plant species was pooled prior to surface sterilization. In total, 30 leaf and 30 root samples were obtained (for details see Table 1).

### Surface sterilization of plant material

Aerial plant parts (shoots and leaves) of 30 the samples were surface-sterilized by serial washing in 70% ethanol for 1 min, 2% sodium hypochlorite for 30 s and 70% ethanol for 1 min, followed by two times immersion in sterile, distilled water for 30 s and once in sterile, diethylpyrocarbonate (DEPC)-treated water. Plant roots were washed with tap water to remove soil. Surface sterilization of roots was performed according to Li et al. (2010), with slight modifications. In this study, 2% sodium hypochlorite and sterile DEPC-treated water were used. To confirm the success of the disinfection procedure, two methods were performed as described previously (Wemheuer et al., 2016). In brief, aliquots of the water used in the final wash step were plated on common laboratory media plates. The plates were incubated in the dark at 25°C for at least 1 week. No growth of microorganisms was observed. In addition, water from the same aliquots was subjected to PCR targeting the bacterial 16S rRNA gene and ITS region of fungal rDNA. No PCR product was detected. These results confirmed that the surface sterilization was successful in eliminating cultivable as well as non-cultivable epiphytic bacteria and fungi as well as potential DNA traces from the plant surfaces. Surface-sterilized plant material was ground to a fine powder in liquid nitrogen using an autoclaved mortar and pestle. Aliquots of the obtained powder were stored at −20°C until DNA extraction.

### Extraction of total community DNA

Total DNA of aerial plant parts and roots was extracted employing the peqGOLD Plant DNA Mini kit (Peqlab, Erlangen, Germany) according to the manufacturer's instructions with two modifications as described previously (Wemheuer et al., 2016). Total environmental DNA of rhizosphere as well as bulk soil samples was extracted employing the PowerSoil® DNA Isolation kit (MoBio Laboratories, Inc., Carlsbad, USA) according to the manufacturer's protocol. DNA concentration of DNA extracts was quantified using a NanoDrop ND-1000 spectrophotometer (NanoDrop Technologies, Wilmington, DE, USA). In total, DNA of 110 samples was subjected to PCR targeting the bacterial 16S rRNA gene and the fungal ITS region.

### Amplification of the 16S rRNA gene

Bacterial endophyte and soil communities were assessed by a nested PCR approach targeting the 16S rRNA gene. For details of the first PCR mixture and the thermal cycling scheme see Wemheuer et al. (2016). In brief, the primers 799f (5′-AACMGGATTAGATACCCKG-3′) (Chelius and Triplett, 2001) and 1492R (5′-GCYTACCTTGTTACGACTT-3′) (Lane, 1991) were used in the first PCR to suppress co-amplification of chloroplast-derived 16S rRNA genes (Chelius and Triplett, 2001). PCR amplification resulted in two PCR products: a bacterial product of approximately 735 bp and a mitochondrial product with approximately 1.1 kbp. Genomic DNA of *Bacillus licheniformis* DSM13 was used as template in the positive control for the bacterial product. Obtained PCR products were subjected to nested PCR.

The V6-V8 region of the 16S rRNA gene was amplified with the primers 968F and 1401R (Nübel et al., 1996) containing MiSeq adaptors (underlined) (MiSeq-968F 5′-TCGTCGGCAGCGTCAGATGTGTATAAGAGACAGAACGCGAAGAACCTTAC-3′; MiSeq- 1401R 5′-GTCTCGTGGGCTCGGAGATGTGTATAAGAGACAGCGGTGTGTACAAGACCC-3′). The PCR mixture (25 μl) contained 5 μl of 5-fold Phusion HF buffer, 200 μM of each of the four deoxynucleoside triphosphates, 4 μM of each primer, 1 U of Phusion high fidelity DNA polymerase (Thermo Scientific, Waltham, MA, USA) and approximately 50 ng of the bacterial product of the first PCR as template. Negative controls were performed using the reaction mixture without template. The following thermal cycling scheme was used: initial denaturation at 98°C for 30 s, 30 cycles of denaturation at 98°C for 15 s, annealing at 53°C for 30 s, followed by extension at 72°C for 30 s. The final extension was carried out at 72°C for 2 min. Three independent PCRs were performed per sample. Obtained PCR products per sample were controlled for appropriate size, pooled in equal amounts, and purified using the peqGOLD Gel Extraction kit (Peqlab). Quantification of the PCR products was performed using the Quant-iT dsDNA HS assay kit and a Qubit fluorometer (Thermo Scientific) as recommended by the manufacturer. Purified PCR products were barcoded using the Nextera XT-Index kit (Illumina, San Diego, USA) and the Kapa HIFI Hot Start polymerase (Kapa Biosystems, Wilmington, USA). The Göttingen Genomics Laboratory determined the sequences of the partial 16S rRNA genes employing the MiSeq Sequencing platform and the MiSeq Reagent Kit v3 (2 × 300 cycles) as recommended by the manufacturer (Illumina). All bacterial samples were sequenced in one single MiSeq run.

### Amplification of the ITS region

The fungal communities in soil and endosphere were assessed by a nested PCR approach targeting the ITS region as described in Wemheuer and Wemheuer (2017). In the first PCR, the primers ITS1-F_KYO2 (5′-TAGAGGAAGTAAAAGTCGTAA-3′) (Toju et al., 2012) and ITS4 (5′- TCCTCCGCTTATTGATATGC-3′) (White et al., 1990) were used to suppress co-amplification of plant-derived ITS regions. The PCR mixture (25 μl) contained: 5 μl of 5-fold Phusion GC buffer, 200 μM of each of the four deoxynucleoside triphosphates, 4 μM of each primer, 5% DMSO, 25 mM MgCl_2_, 0.5 U of Phusion High Fidelity DNA polymerase (Thermo Scientific) and approximately 10 ng DNA sample as template. Negative controls were performed using the reaction mixture without template. The following thermal cycle scheme was utilized: initial denaturation at 98°C for 30 s followed by 6 cycles of denaturation at 98°C for 15 s, annealing at 53°C for 30 s decreasing 0.5°C in each cycle, followed by extension at 72°C for 30 s and 29 cycles of denaturation at 98°C for 15 s, annealing at 50°C for 30 s, followed by extension at 72°C for 30 s. The final extension was carried out at 72°C for 2 min. Obtained PCR products were subjected to nested PCR.

The ITS2 region was subsequently amplified as described for the first PCR using approximately 50 ng product of the first PCR and the primers ITS3_KYO2 (Toju et al., 2012) and ITS4 (White et al., 1990) containing the MiSeq adaptors (underlined): MiSeq-ITS3_KYO2 (5′-TCGTCGGCAGCGTCAGATGTGTATAAGAGACAGGATGAAGAACGYAGYRAA-3′) and MiSeq-ITS4 (5′-GTCTCGTGGGCTCGGAGATGTGTATAAGAGACAGTCCTCCGCTTATTGATATGC-3′). Purification and quantification of obtained PCR products were performed as described for the bacterial PCR products. Three independent PCRs were performed per sample and obtained PCR products were pooled in equal amounts. Purified PCR products were barcoded using the Nextera XT-Index kit (Illumina) and the Kapa HIFI Hot Start polymerase (Kapa Biosystems). The Göttingen Genomics Laboratory determined the sequences of the ITS2 region employing the MiSeq Sequencing platform and the MiSeq Reagent Kit v3 (2 × 300 cycles) as recommended by the manufacturer (Illumina). All fungal samples were sequenced in one single MiSeq run.

### Processing of bacterial and fungal datasets

The Trimmomatic version 0.32 (Bolger et al., 2014) was initially used to truncate low quality reads if quality dropped below 20 in a sliding window of 10 bp. Datasets were subsequently processed with Usearch version 8.0.1623 (Edgar, 2010) as described in Wemheuer and Wemheuer (2017). In brief, paired-end reads were merged and quality-filtered. Filtering included the removal of low quality reads (maximum number of expected errors >1 and more than 1 ambitious base, respectively) and those shorter than 200 bp. Processed sequences of all samples were joined and clustered in operational taxonomic units (OTUs) at 3% genetic divergence using the UPARSE algorithm implemented in Usearch. A *de novo* chimera removal was included in the clustering step. All OTUs consisting of one single sequence (singletons) were removed. Afterwards, remaining chimeric sequences were removed using the Uchime algorithm in reference mode with the most recent RDP training set (version 15) as reference dataset (Cole et al., 2009) for bacteria and the most recent Uchime reference data (version 7.0) obtained from the UNITE database (Kõljalg et al., 2013) for fungi, respectively. Afterwards, OTU sequences were taxonomically classified using QIIME (Caporaso et al., 2010) by BLAST alignment against the SILVA database (SILVA SSURef 128 NR) and the QIIME release of the UNITE database (version 7.1; August 2016), respectively. All non-bacterial or non-fungal OTUs were removed based on their taxonomic classification in the respective database. Subsequently, processed sequences were mapped on OTU sequences to calculate the distribution and abundance of each OTU in every sample. Final OTUs tables for bacteria and fungi are provided as Tables S3, S4, respectively. Only OTUs occurring in more than two samples were considered for further statistical analysis.

### Data analysis

All data analyses were conducted in R version 3.3.1 (R Core Team, 2016). R code used for statistical analysis is provided as Supplementary Data Sheet 1. Differences were considered as statistically significant with *P* ≤ 0.05 and as marginally significant with *P* ≤ 0.1. All bacterial and fungal samples with >276 bacterial and >20 fungal sequences, respectively, were removed prior to statistical data analysis.

Alpha diversity indices (Richness, Shannon index of diversity, effective number of species, and Michaelis Menten Fit) were calculated in the vegan package version 2.4 (Oksanen et al., 2016) and the drc package (Ritz and Streibig, 2005). In brief, OTU tables were rarefied using the *rrarefy* function to 276 bacterial and 20 fungal sequences. Richness and diversity were calculated using the *specnumber* and *diversity* function, respectively. The effective number of species was calculated from the diversity according to Jost (2006). The Michaelis-Menten Fit was calculated as described previously (Wemheuer et al., 2012). All alpha diversity indices were calculated 10 times. The average from each iteration was used for further statistical analysis. Final tables containing bacterial and fungal richness and diversity are provided as Tables S5 and S6, respectively.

Differences in richness and diversity as well as measured edaphic and plant properties between the cropping regimes were tested by Kruskal-Wallis test, respectively. We analyzed the effect of cropping regimes on diversity and richness of fungi and bacteria in all investigated compartments separately to avoid spatial pseudoreplication. Differences between single treatments were tested by pairwise Wilcoxon test without *P*-values correction. To analyze possible effects of plant compartment on richness or diversity, a repeated measures ANOVA (Crawley, 2007) was conducted as communities of different parts of the same plant were compared with each other (spatial pseudoreplication).

Differences in community structure were investigated by permutational multivariate analysis of variance (PERMANOVA) based on Bray-Curtis distance matrices using the *vegdist* and *adonis* function within the vegan package. Bacterial and fungal communities were tested separately. In addition, OTU table used for beta-diversity analysis were rarefied to 276 bacterial and 20 fungal sequences, respectively. Differences with regard to crop species were tested after exclusion of bulk soil samples of the intercropping regimes as the communities in these samples are most probably influenced by both plant species. Differences in community structure were visualized using the *metaMDS* function within the vegan package. Differences of abundant bacterial genera and fungal species were tested by pairwise *t*-test without *p*-value adjustment.

Correlation-based co-occurrence patterns were calculated with respect to cropping regimes to investigate the interactions between fungi and bacteria in soil and endosphere. Therefore, bacterial and fungal OTU tables were combined resulting in a total of 98 samples (20 bulk soil samples, 29 rhizosphere samples, 26 root samples, and 23 leaf samples). One subset contained all samples from one cropping regime in one plant compartment of a single plant species. Pairwise correlation based on Spearman's rho were calculated using the *cor.test* function in R and the number of significant positive and significant negative correlations were counted. Positive correlations were considered as two taxa co-occurring or cooperation between the two taxa. Negative correlations were considered as two taxa avoiding each other or competition between the two taxa.

To identify OTUs highly associated to cropping regime with respect to plant species and plant compartment, multipattern analyses were applied. For that purpose, fungal and bacterial OTUs were investigated using the *multipatt* function from the IndicSpecies package (De Cáceres and Legendre, 2009). The resulting biserial coefficients (*R*) of each OTU with a particular regime were corrected for unequal sample size using the function *r.g* (Tichy and Chytry, 2006). As a single taxon can occupy a certain niche in several cropping systems, it is necessary to consider all possible combinations to detect these associations (De Cáceres et al., 2010).

### Sequence data deposition

Sequence data were deposited in the sequence read archive (SRA) of the National Center for Biotechnology Information (NCBI) under accession number SRA419369.

## Results and discussion

### Soil characteristics and plant growth

Soil pH-values were constant among all soil samples (pH_water_ = 6.82 ± 0.13; pH_KCl_ = 6.55 ± 0.09) with no significant differences between the different cropping regimes (Table S1). Soil moisture varied between 13.7 and 38.4% (Table S1), being significantly lower in bulk soil samples of WM than in bulk soil samples of FBM (Table 2). The C:N ratio in bulk soil samples of the cropping regime MI was significantly higher compared to the other cropping regimes. The C:N ratio explains the ability to use soil carbon and nitrogen for microbial processes such as the decomposition of soil organic matter (Wardle, 1992). We speculate that the higher C:N ratio observed in intercropping regime MI might be related to a smaller distance between the cereal and the legume root system, which influences the N transfer from the legume to wheat (Fujita et al., 1992).

**Table 2:** **Edaphic parameters (means ± SE)**.

	**Soil moisture (%)**	**C_total_ (%)**	**N_total_ (%)**	**C:N ratio**
**BULK SOIL**				
FBM	31.22 ± 6.25a	8.42 ± 2.35a	0.18 ± 0.05a	46.33 ± 1.09a
WM	18.14 ± 4.03c	6.33 ± 0.54a	0.14 ± 0.01a	45.11 ± 1.26a
RI	21.54 ± 3.61b,c	7.68 ± 2.15a	0.17 ± 0.04a	46.47 ± 1.08a
MI	24.99 ± 2.19a,b,c	7.83 ± 1.03a	0.17 ± 0.02a	47.41 ± 0.37b
**FABA BEAN RHIZOSPHERE**				
FBM	34.43 ± 3.16a	10.50 ± 1.02a	0.22 ± 0.03a	48.24 ± 1.68a
RI	29.12 ± 4.11a	13.76 ± 2.63a	0.27 ± 0.05a	50.13 ± 1.07a
MI	26.39 ± 6.33a	11.89 ± 2.17a	0.24 ± 0.05a	49.99 ± 2.20a
**WHEAT RHIZOSPHERE**				
WM	22.33 ± 2.99a	10.86 ± 1.66a	0.22 ± 0.03a	50.29 ± 2.25a
RI	26.44 ± 3.56a	12.08 ± 2.26a	0.24 ± 0.05a	50.92 ± 1.22a
MI	24.86 ± 6.14a	9.48 ± 1.55a	0.18 ± 0.03a	51.43 ± 1.55a

*Different letters within columns indicate significant differences with P ≤ 0.05. C_total_, total soil organic carbon; N_total_, total soil nitrogen; FBM, faba bean in monoculture; WM, wheat in monoculture; MI, mixed intercropping; RI, row intercropping*.

To analyze the effect of cropping regime on plant growth and yield, aboveground as well as root biomass were measured (Table S2). A significantly higher average root biomass was observed for faba bean and wheat plants grown in rows (RI) compared to those grown in monocultures or in intercropping regime MI (Table 3). One possible explanation is that there is a higher intraspecific competition in RI as environmental stresses increase the relative weight of roots compared to shoots (Eghball and Maranville, 1993). In the present study, the shoot/root ratio for faba bean monocultures was significantly higher than that of faba bean under intercropping regime RI. We speculate that this is caused by a higher above- and belowground competition between faba bean and wheat for space, nutrients, and water (Mariotti et al., 2009). In addition, these results might be related to interspecific competition and facilitation that act on the crop plants in intercropping systems simultaneously (Ghosh et al., 2006; Mariotti et al., 2009). We suggest that the differences observed for root biomass of plants under MI and RI are related to the fact, that competition and facilitation effects between plants can be altered through different row arrangements, inter-row spacing, sowing time, plant densities, and proportions of plants (Fujita et al., 1992; Mariotti et al., 2009).

**Table 3:** **Growth characteristics of faba bean and wheat plants**.

	**Height (cm)**	**Aboveground biomass (g)**	**Water content (%)**	**Root biomass (g)**	**Shoot/root ratio**
**FABA BEAN**					
FBM	21.40 ± 1.84a	4.83 ± 1.16a	90.47 ± 0.55a	2.34 ± 0.49a	2.11 ± 0.52a
RI	18.80 ± 1.69a	3.27 ± 0.52a	87.92 ± 1.66b	2.66 ± 0.65b	1.29 ± 0.32b
MI	19.26 ± 2.68a	3.72 ± 1.40a	87.10 ± 2.49b	1.98 ± 0.69a	1.92 ± 0.43ab
**WHEAT**					
WM	38.78 ± 1.12a	1.36 ± 0.18a	84.58 ± 4.01a	1.94 ± 0.48a	0.76 ± 0.25a
RI	40.30 ± 3.00a	1.64 ± 0.27a	82.40 ± 2.02a	2.78 ± 1.22b	0.71 ± 0.29a
MI	39.76 ± 1.40a	1.68 ± 0.37a	84.72 ± 1.12a	2.05 ± 0.49a	0.86 ± 0.22a

*Different letters in columns indicate statistically significant differences between groups (P ≤ 0.05, means ± SE). The above- and belowground biomass per plant (g) is shown. For height, approximately 10 faba bean and 20 wheat plants in intercropping regimes and approximately 20 plants of monocultured faba bean and wheat plants were measured. FBM, faba bean in monoculture; WM, wheat in monoculture; MI, mixed intercropping; RI, row intercropping*.

### Bacterial and fungal communities are dominated by a few phyla

The response of bacterial and fungal communities of faba bean and wheat toward cropping regimes was assessed by Illumina (MiSeq) sequencing targeting the bacterial 16S rRNA gene and the fungal ITS region, respectively. Sequencing of bacterial 16S rRNA and fungal ITS gene amplicons from all samples resulted in 9,428,318 and 6,416,722 paired reads, respectively (Table S7). After removal of low quality reads, PCR artifacts (chimeras) and plant-derived contaminations, a total of 897,824 and 282,209 high-quality reads were obtained for bacteria and fungi, respectively. Sequence numbers per sample varied between 4 to 30,936 (average 8,313) for bacteria and 2 to 48,421 (average 2,637) for fungi. We attribute the high loss of fungal sequences to an average low quality of the reverse reads and the high plant-derived contamination (Table S7).

Obtained sequences were grouped into 695 bacterial and 188 fungal OTUs (Tables S3, S4). Richness (number of observed OTUs) and diversity (Shannon indices) for bacterial communities ranged from 8.1 to 70.7 and from 1.32 to 3.23, respectively (Table 4). For fungal communities, richness, and Shannon indices ranged from 7.0 to 12.7 and from 1.60 to 2.41. Effective number of species ranged from 3.7 to 25.2 for bacteria and from 5.0 to 11.1 for fungi. Although samples were rarefied to low sequencing numbers (bacteria = 276 sequences, fungi = 20 sequences), calculated Michaelis-Menten Fit confirmed that the majority of bacterial and fungal communities was recovered by the surveying effort (Tables S5, S6). All OTUs were classified below phylum level.

**Table 4:** **Bacterial and fungal richness and diversity with regard to plant compartment and cropping regimes**.

	**Bacteria**			**Fungi**		
	**Richness**	**Diversity**	**Effective no. of species**	**Richness**	**Diversity**	**Effective no. of species**
**FABA BEAN BULK SOIL**						
FBM	32.2 ± 4.1a	1.62 ± 1.50ab	5.0 ± 0.6ab	9.8 ± 1.5a	2.05 ± 2.03ab	7.8 ± 1.4ab
RI	33.6 ± 4.2a	1.54 ± 1.47a	4.7 ± 0.6a	9.2 ± 1.2a	1.98 ± 1.96a	7.2 ± 1.4a
MI	69.1 ± 4.9a	3.23 ± 2.83b	25.2 ± 2.2b	11.2 ± 1.9a	2.24 ± 2.24b	9.4 ± 1.8b
**WHEAT BULK SOIL**						
WM	56.0 ± 4.4a	2.62 ± 2.33ab	13.7 ± 1.4ab	10.6 ± 1.5a	2.13 ± 2.08a	8.4 ± 1.6a
RI	33.6 ± 4.2a	1.54 ± 1.47a	4.7 ± 0.6a	9.2 ± 1.2a	2.07 ± 0.16a	7.2 ± 1.4a
MI	69.1 ± 4.9a	3.23 ± 2.83b	25.2 ± 2.2b	11.2 ± 1.9a	2.25 ± 0.11a	9.4 ± 1.8a
**FABA BEAN RHIZOSPHERE**						
FBM	64.9 ± 5.3a	2.91 ± 2.61a	18.3 ± 1.9a	11.4 ± 1.6a	2.21 ± 2.19a	9.1 ± 1.8a
RI	51.1 ± 4.2a	2.34 ± 2.12a	10.4 ± 1.4a	10.6 ± 1.5a	2.18 ± 2.16a	8.8 ± 1.4a
MI	52.3 ± 4.6a	2.33 ± 2.27a	10.3 ± 1.2a	10.7 ± 1.7a	2.18 ± 2.17a	8.8 ± 1.8a
**WHEAT RHIZOSPHERE**						
WM	45.2 ± 3.9a	2.19 ± 2.05a	8.9 ± 0.9a	10.8 ± 1.7a	2.20 ± 2.18a	9.0 ± 1.8a
RI	47.7 ± 4.6ab	2.03 ± 1.93a	7.6 ± 1.0a	9.9 ± 1.5a	2.04 ± 2.03a	7.7 ± 1.5a
MI	70.7 ± 5.9b	3.01 ± 2.87a	20.2 ± 2.8a	11.4 ± 1.6a	2.24 ± 2.23a	9.4 ± 1.6a
**FABA BEAN ROOTS**						
FBM	52.7 ± 3.7a	2.76 ± 2.71a	15.8 ± 1.2a	7.7 ± 1.7a	1.70 ± 1.55a	5.5 ± 1.3a
RI	38.2 ± 3.4ab	2.24 ± 2.10a	9.4 ± 0.8a	11.9 ± 0.4a	2.18 ± 2.18a	8.9 ± 1.7a
MI	24.5 ± 2.4b	1.87 ± 1.52a	6.5 ± 0.4a	12.2 ± 1.3a	2.29 ± 2.28a	9.9 ± 1.6a
**WHEAT ROOTS**						
WM	47.5 ± 3.6a	2.50 ± 2.45a	12.2 ± 1.1a	9.2 ± 1.1a	2.01 ± 1.78a	7.4 ± 1.0a
RI	42.3 ± 4.4a	2.37 ± 2.28a	10.7 ± 1.3a	10.0 ± 1.5a	2.05 ± 2.05a	7.8 ± 1.5a
MI	48.5 ± 3.6a	2.61 ± 2.39a	13.7 ± 1.4a	11.0 ± 1.5a	2.13 ± 2.11a	8.4 ± 1.9a
**FABA BEAN LEAVES**						
FBM	17.3 ± 2.1a	1.47 ± 1.34a	4.3 ± 0.3a	11.9 ± 1.3ab	2.30 ± 2.30ab	10.0 ± 1.4ab
RI	15.3 ± 1.2a	1.32 ± 1.26a	3.7 ± 0.2a	10.1 ± 1.2a	1.98 ± 1.97a	7.2 ± 1.3a
MI	16.1 ± 2.0a	1.88 ± 1.77a	6.5 ± 0.5a	12.7 ± 0.8b	2.41 ± 2.39b	11.1 ± 0.9b
**WHEAT LEAVES**						
WM	9.5 ± 1.0a	1.47 ± 1.31a	4.3 ± 0.2a	8.7 ± 1.6a	1.88 ± 1.82a	6.5 ± 1.5a
RI	8.1 ± 0.8a	1.34 ± 1.30a	3.8 ± 0.2a	7.0 ± 1.6a	1.60 ± 1.39a	5.0 ± 1.1a
MI	10.4 ± 0.9a	1.73 ± 1.70a	5.6 ± 0.3a	9.2 ± 1.5a	1.99 ± 1.90a	7.3 ± 1.2a

*Diversity is expressed as Shannon values and richness is based on the number of observed OTUs. Different letters in columns indicate statistically significant differences between the cropping regimes for each plant compartment (P ≤ 0.05, means ± SE). FBM, faba bean in monoculture; WM, wheat in monoculture; MI, mixed intercropping; RI, row intercropping. There was a marginal effect of cropping regimes on bacterial richness in bulk soil of faba bean samples (P = 0.062), and on bacterial diversity and the effective number of species in faba bean roots (P = 0.079)*.

**Figure 3:**
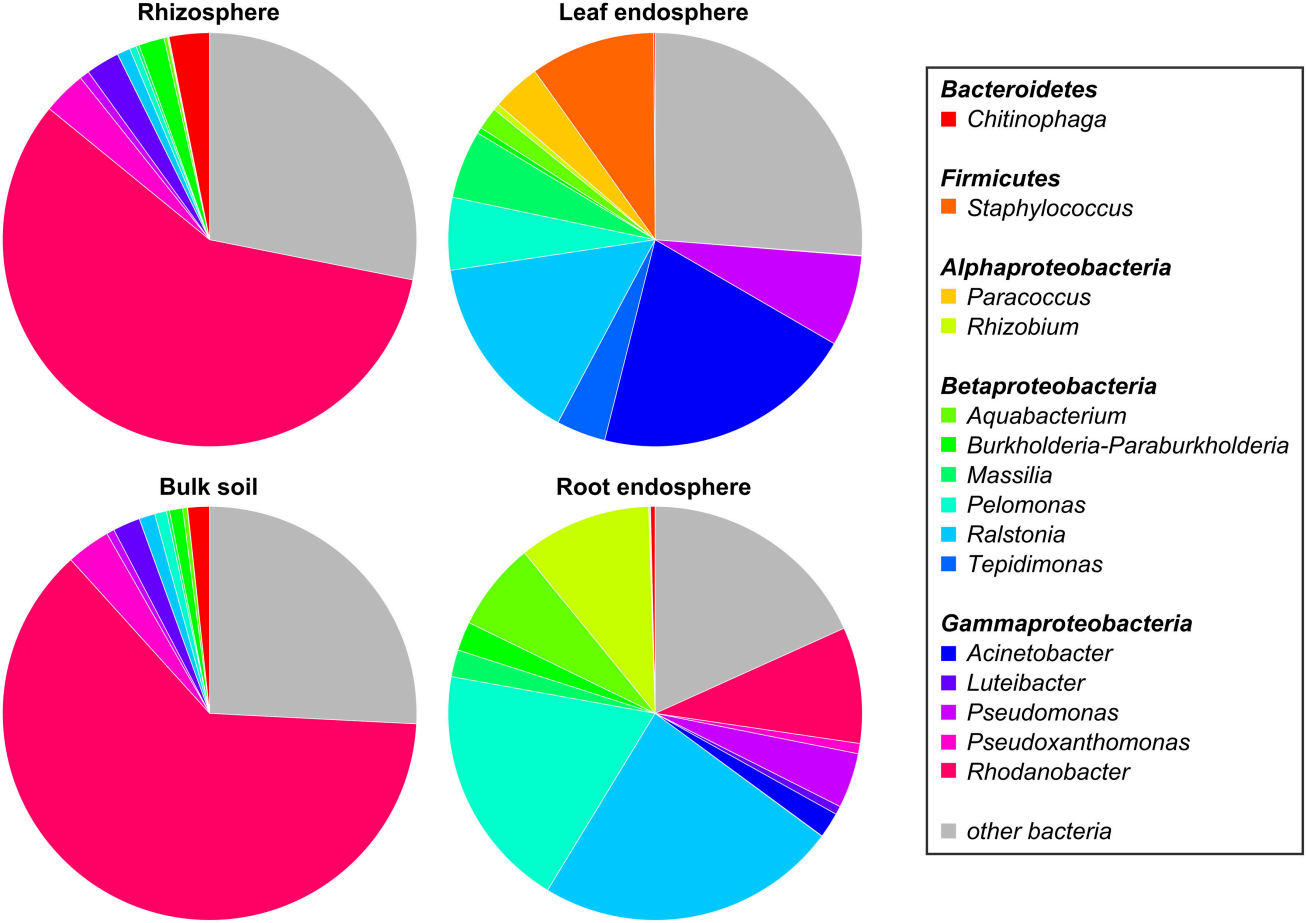
**Abundant bacterial phyla, proteobacterial classes, and genera, derived from the different plant compartments**. Only groups with an average abundance >1% in at least one of the investigated plant species are shown. Mean relative abundances of each taxa were calculated based on relative abundances calculated for each sample.

**Figure 4:**
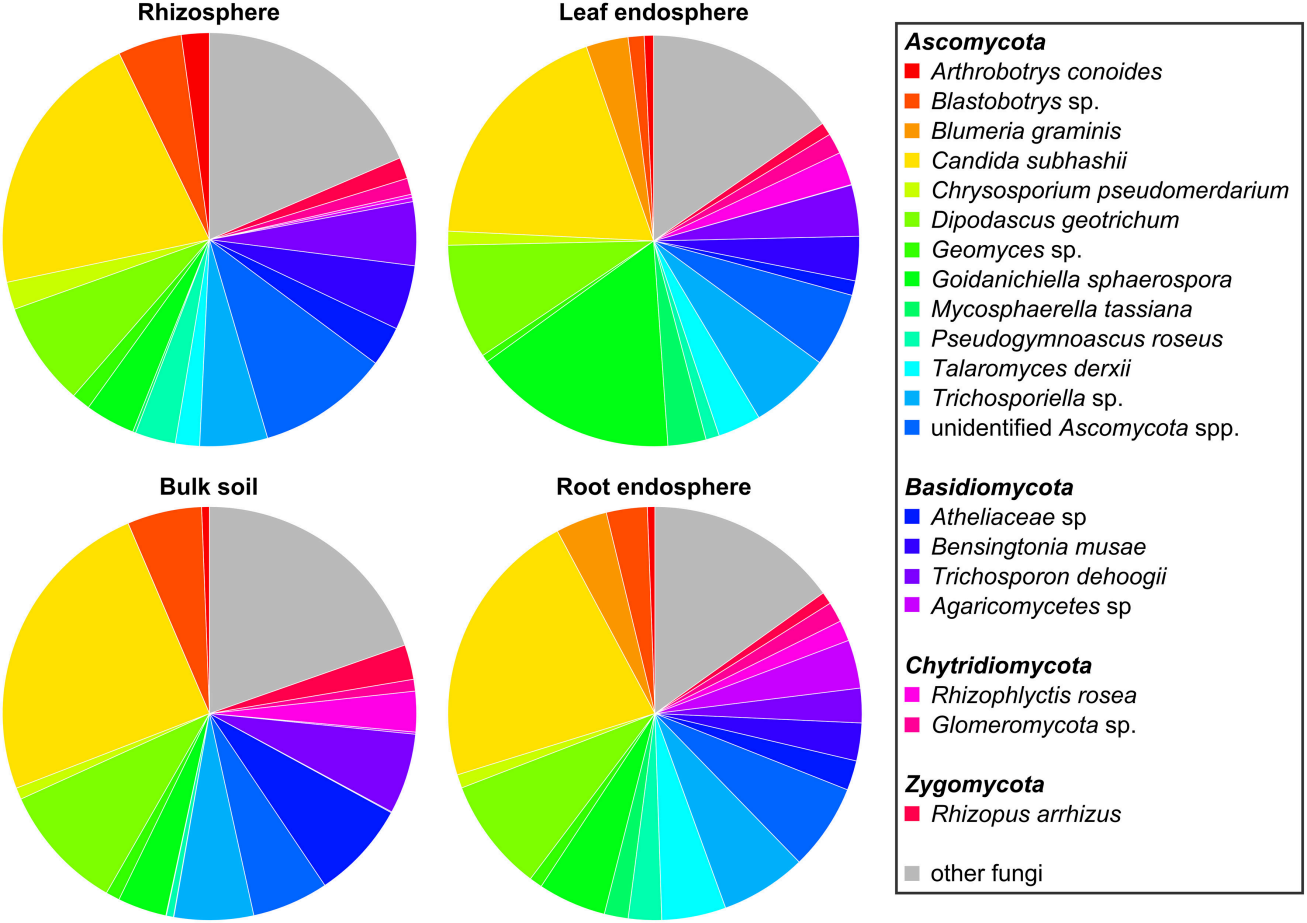
**Abundant fungal phyla and species in the investigated plant species, derived from the different plant compartments**. Only groups with an average abundance >1% in at least one of the investigated plant species are shown. Mean relative abundances of each taxa were calculated based on relative abundances calculated for each sample.

The five dominant bacterial phyla (>1% of all sequences across all samples) were *Proteobacteria* (82.73%), *Actinobacteria* (5.25%), *Firmicutes* (5.21%), *Bacteroidetes* (2.37%), and *Acidobacteria* (1.20%) (Figure 3, Table S3). Fungi were represented by the abundant phyla *Ascomycota* (74.70%), *Basidiomycota* (14.34%), *Chytridiomycota* (2.32%), *Zygomycota* (1.76%), and *Glomeromycota* (1.38%) (Figure 4, Table S4). The abundant bacterial and fungal phyla were present in all samples and accounted for 96.76 and 92.82%, respectively, of all sequences analyzed in this study. These results are in line with previous studies investigating plant-associated bacterial and fungal communities (Bulgarelli et al., 2015; Detheridge et al., 2016; Wemheuer et al., 2017).

At genus level, *Rhodanobacter* (29.64%) was predominant across all samples with higher abundances in soil samples (Figure 3). Other abundant bacterial genera observed in this study were *Acinetobacter* (6.50%), *Ralstonia* (11.06%), *Pelomonas* (7.08%), *Pseudomonas* (3.52%), *Rhizobium* (2.97%), *Staphylococcus* (2.84%), *Aquabacterium* (2.49%), *Massilia* (2.23%), *Pseudoxanthomonas* (1.77%), and *Chitinophaga* (1.30%). The predominance of *Rhodanobacter* and the high abundance of *Ralstonia* are not in line with a recent study investigating soil bacterial communities (Kaiser et al., 2016). In contrast, very high abundances of *Rhodanobacter* in fertilized soil samples derived from a temperate forest in the Hainich National Park (Germany) were observed (Pfeiffer, 2013). We speculate that the high abundances of *Rhodanobacter* and *Ralstonia* are related to the commercial potting soil used as these genera were isolated from different potting media products in a recent study (Al-Sadi et al., 2016).

The predominant fungal OTU observed in the present study belonged to *Dipodascus geotrichum*. Abundant fungal species were, for example, *Candida subhashii* (21.36%), *D. geotrichum* (8.93%), *Goidanichiella sphaerospora* (7.68%), *Trichosporon dehoogii* (4.36%), one member of *Blastobotrys* sp. (3.62%), *Bensingtonia musae* (3.13%), *Blumeria graminis* (1.98%), and *Arthrobotrys conoides* (1.07%). The predominance of *C. subhashii* supports the results of de Souza et al. (2016). They showed that members of the genus *Candida* accounted for up to 9.4% of the relative abundances in sugarcane stalks and belonged to the core microbiome. *Candida subhashii* was considered as human pathogenic yeast as it has been isolated from a patient sample (Adam et al., 2009). Nonetheless, only one case report of *C. subhashii* infections exists so far (Adam et al., 2009). In a recent study, *C. subhashii* was repeatedly isolated from commercially available potting soil as well as from soil samples indicating that this yeast is a common soil fungus (Hilber-Bodmer et al., 2017). In addition, the yeast occurred in large concentrations in potting substrates, which might explain the high abundance of this yeast observed in the present study.

Overall, predominant endophytic bacterial genera in roots and leaves differed in their distribution (Figure 3). This supports the results of Robinson et al. (2016) who showed that leaf and root endophyte communities of wheat differed in abundance and structure. The authors concluded that below-and aboveground endosphere represent two distinct ecological niches for bacteria in the plant microbiome creating different conditions for colonization and establishment of bacterial endophytes. In the present study, soil and endophyte compartments were dominated by different bacterial genera. Moreover, similar patterns of fungal species were observed for rhizosphere and bulk soil as well as for root endosphere (Figure 4). However, a direct comparison between endosphere and soil communities should be treated with caution as two different DNA extraction methods were used for endophyte and soil communities.

### Cropping regime influenced microbial diversity and richness

We compared bacterial as well as fungal richness (number of observed OTUs), diversity (represented by the Shannon index H'), and the number of effective species between the four cropping regimes. Each plant compartment was analyzed separately to prevent spatial pseudoreplication. We detected differences in bacterial diversity and richness between the four cropping regimes (Table 4). Bacterial diversity and the effective number of bacterial species were significantly higher in bulk soil samples of wheat and faba bean grown in MI compared to RI. Bacterial richness was significantly lower in roots of faba bean under MI compared to faba bean monoculture while bacterial diversity was only marginally affected. This is in accordance with a previous study showing that intercropping with maize did not affect bacterial diversity of soybean root endophytes (Zhang et al., 2011).

In the present study, bacterial richness in the rhizosphere of wheat grown in MI was significantly higher compared to that in rhizosphere of wheat monoculture, whereas bacterial diversity was not affected. Contrary, Yang et al. (2016) observed that bacterial diversity in rhizosphere soil of 10 common spring crops in North China was higher under intercropping than under monoculture regime. However, our results are in accordance with a previous study analyzing the effects of intercropping with maize and *Rhizobium* inoculation on rhizosphere bacterial diversity (Zhang et al., 2010). Here, intercropping did not affect bacterial diversity.

Cropping regimes did not affect fungal richness and diversity with two exceptions (Table 4): fungal diversity, richness, and effective number of species in leaf endosphere of faba bean grown in RI were significantly lower than in MI. In addition, a lower fungal diversity and effective number of species were observed in bulk soil of faba bean under RI compared to intercropping regime MI. Bacterial and fungal richness and diversity in bulk soil samples of faba bean and wheat did not differ between plants derived from monocultures and intercropping regimes. This is not in line with a recent study of Venter et al. (2016). They found a positive effect of an increasing crop diversity on soil microbial richness and diversity. A possible reason for the increased fungal diversity in bulk soil of MI is the higher C/N ratio measured in these samples. This supports the results of Högberg et al. (2007) who showed that the fungal biomass decreased with decreasing soil C:N ratio. In addition, they found that the abundance of bacterial community in soil was significantly and negatively related to soil C:N ratio, which is in contrast to our results.

We speculate that the contrasting effects of cropping regimes on microbial diversity observed here and in other studies are related to differences in plant species, root exudates, plant age, and soil type, as these factors influence microbial diversity (Berg and Smalla, 2009; Zhang et al., 2011). Another possible explanation for the contrasting effects is that synergistic and antagonistic interactions occurred between plants growing in mixed cultures (Wang et al., 2012). This might affect microbial diversity and richness in a different way as plant diversity and proportion differed in the various cropping regimes.

### Effect of cropping regimes on microbial community structure is determined by crop species and plant compartment

**Figure 5:**
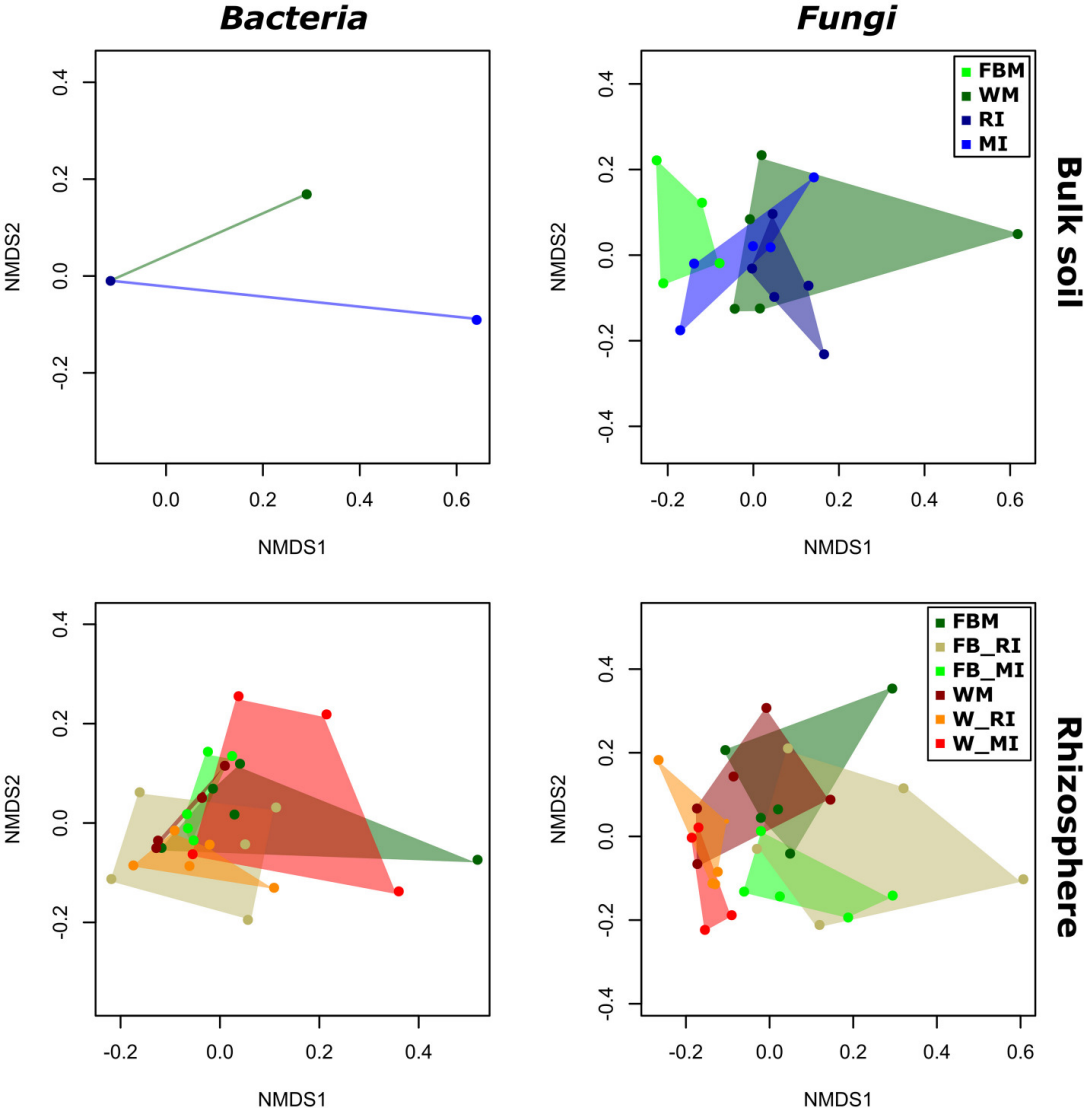
**Response of bacterial and fungal communities in bulk and rhizosphere soil toward cropping regimes**. NMDS ordination of bacterial and fungal soil communities color-coded by the respective cropping regime. Ordination is based on Bray-Curtis dissimilarities between samples. Note that two mixed intercropping samples and two wheat monocropping samples of bulk soil bacteria formed two clusters distinct from each other and from all other samples. Consequently, several samples are masked and cannot be distinguished from each other. FBM, all samples derived from faba bean monoculture; WM, all samples derived from wheat monoculture; FB_MI, faba bean samples from mixed intercropping; FB_RI, faba bean samples from row intercropping; W_MI, wheat samples from mixed intercropping; W_RI, wheat samples from row intercropping; MI, samples from mixed intercropping; RI, samples from row intercropping.

**Figure 6:**
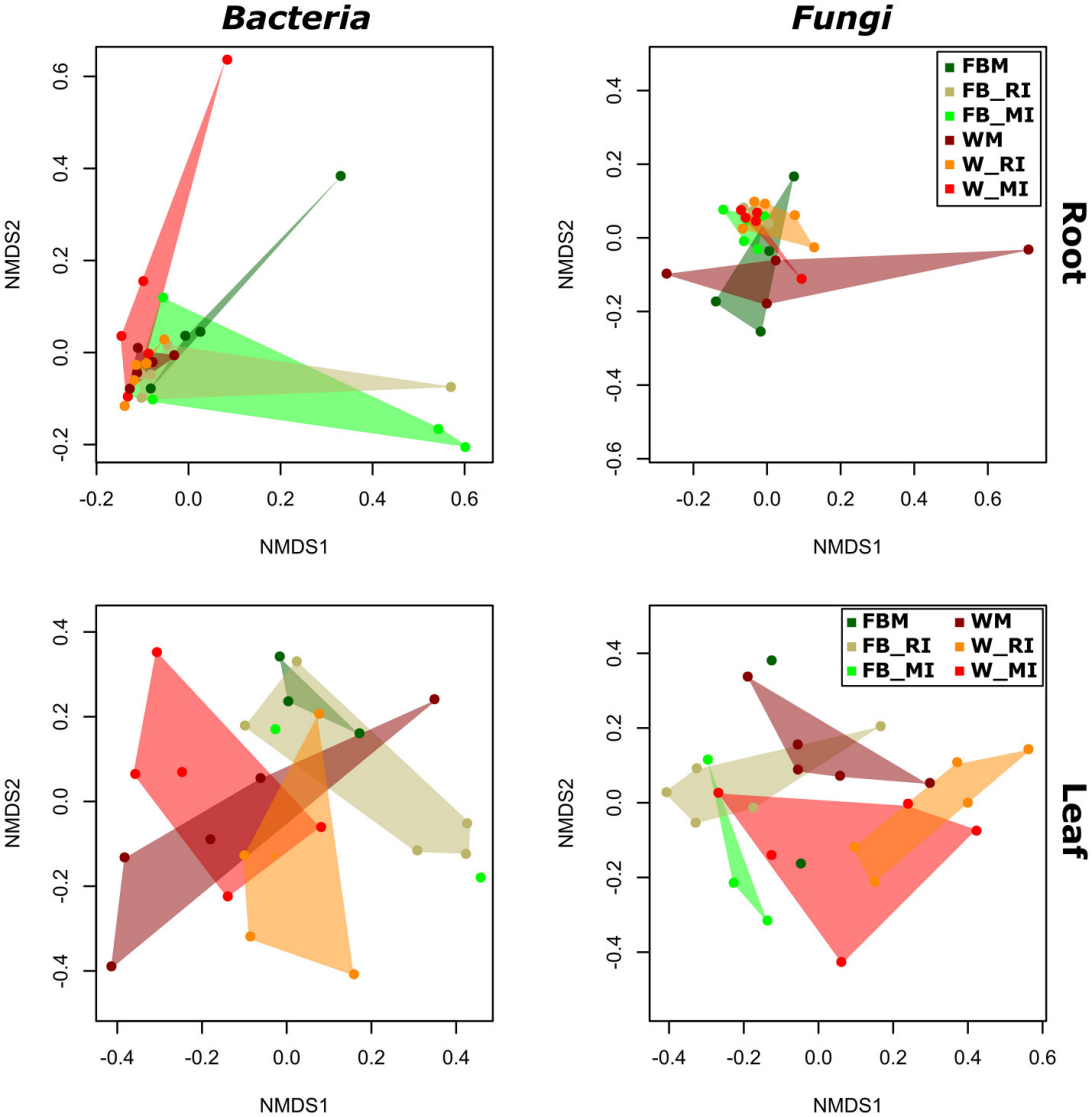
**Response of bacterial and fungal communities in leaf and root endosphere toward cropping regimes**. NMDS ordination of bacterial and fungal endophyte communities color-coded by the respective cropping regime. Ordination is based on Bray-Curtis dissimilarities between samples. FBM, all samples derived from faba bean monoculture; WM, all samples derived from wheat monoculture; FB_MI, faba bean samples from mixed intercropping; FB_RI, faba bean samples from row intercropping; W_MI, wheat samples from mixed intercropping; W_RI, wheat samples from row intercropping; MI, samples from mixed intercropping; RI, samples from row intercropping.

To identify the influence of the different cropping regimes on microbial community structures, multivariate statistics (non-metric multidimensional scaling; NMDS) were performed. Distinct clustering with respect to cropping regimes was observed for bacterial and fungal communities only in few plant compartments (Figures 5, 6). For example, fungal communities in bulk soil samples from the cropping regimes FBM and WM differed (Figure 5). In addition, bacterial leaf endophytes of monocropped faba bean and wheat grown under MI formed distinct clusters (Figure 6). We further analyzed the influence of cropping regimes on microbial community profiles by PERMANOVA. Cropping regimes significantly influenced fungal and bacterial communities in bulk soil of faba bean, explaining approximately 25 and 34% of the variance in the dataset (Table 5). Bacterial community structure in the wheat rhizosphere was significantly affected by cropping regime. Here, intercropping explained more than 28% of the variance. Moreover, cropping regimes significantly altered fungal communities in rhizosphere soil and root endosphere, explaining more than 21 and 25% of the variance, respectively.

**Table 5:** **Effect of cropping regimes, crop species, and plant compartment on bacterial and fungal community structures**.

	**Bacteria**				**Fungi**			
	**Faba bean**		**Wheat**		**Faba bean**		**Wheat**	
	***R*^2^ (%)**	***P***	***R*^2^ (%)**	***P***	***R*^2^ (%)**	***P***	***R*^2^ (%)**	***P***
**BULK SOIL**								
Cropping regime	**34.03**	**0.013**	25.37	0.118	**25.44**	**0.005**	20.00	0.058
Mo vs. RI	11.94	0.315	**26.33**	**0.030**	**25.91**	**0.005**	16.36	0.064
Mo vs. MI	**28.94**	**0.024**	7.64	0.508	13.65	0.194	11.23	0.438
RI vs. MI	**28.23**	**0.032**	**28.94**	**0.029**	**20.02**	**0.048**	20.02	0.054
**RHIZOSPHERE**								
Cropping regime	18.22	0.198	**28.46**	**0.023**	**21.86**	**0.02**	**26.4**	**0.013**
Mo vs. RI	11.15	0.41	20.28	0.085	15.17	0.141	16.40	0.124
Mo vs. MI	14.09	0.173	**25.12**	**0.047**	**21.65**	**0.019**	20.71	0.076
RI vs. MI	17.70	0.127	**26.10**	**0.042**	17.23	0.072	**23.71**	**0.036**
**ROOT**								
Cropping regime	16.87	0.343	17.42	0.206	**27.6**	**0.013**	**25.82**	**0.012**
Mo vs. RI	10.83	0.501	4.87	0.915	**27.44**	**0.043**	**22.21**	**0.027**
Mo vs. MI	19.83	0.183	14.11	0.221	**20.78**	**0.023**	**26.26**	**0.047**
RI vs. MI	8.37	0.317	16.26	0.149	20.54	0.154	14.33	0.245
**LEAF**								
Cropping regime	25.22	0.36	17.71	0.10	28.4	0.145	18.91	0.151
Mo vs. RI	24.64	0.20	17.65	0.068	23.54	0.144	20.21	0.067
Mo vs. MI	28.72	0.30	10.96	0.568	30.31	0.30	15.46	0.158
RI vs. MI	7.41	0.455	13.28	0.184	16.76	0.298	9.07	0.614
Crop species^*^	1.7	0.127	1.7	0.127	**3.1**	**0.003**	**3.1**	**0.003**
Compartment^*^	**46.3**	**0.001**	**46.3**	**0.001**	**9.7**	**0.001**	**9.7**	**0.001**
Crop species/compartment	**50.5**	**0.001**	**52.4**	**0.001**	**14.1**	**0.002**	**16.5**	**0.001**

*Results of the permutational multivariate analysis of variance (PERMANOVA) for the different cropping regimes. Statistically significant differences (P ≤ 0.05) between the cropping regimes for each plant compartment are written in bold. Marginally significant differences with P ≤ 0.1 are underlined. Mo, Monoculture; MI, mixed intercropping; RI, row intercropping*.

* *The effect of crop species and plant compartment was analyzed for both crop plants*.

Furthermore, we analyzed the effect of cropping regimes on bacterial and fungal communities in each plant compartment (Table 5). We found crop species-specific and plant compartment-specific responses of fungal and bacterial communities toward the cropping regimes. Bacterial and/or fungal communities in bulk soil samples of both crop species showed distinct community structures under the cropping regimes RI and MI. Bacterial community structures in bulk soil samples of monocultured wheat or faba bean differed significantly with those of RI or MI, respectively. The results for soil bacteria are in line with a study investigating the effect of intercropping on bacterial communities (Zhang et al., 2010) in which bacterial communities in intercropped soil were different from those of monoculture soils. We hypothesize that the results of the present study are related to differences in C:N ratio and soil moisture determined in bulk soil samples. As already shown in other studies, these parameters are strong drivers of soil microbial community composition (Högberg et al., 2007; Kaiser et al., 2016).

In the present study, cropping regimes only marginally affected bacterial endophyte community structure in wheat leaves (Table 5). The influence of cropping regimes on fungal endophytes in roots of both faba bean and wheat was more pronounced: the community structure differed significantly between monocultured and intercropped plants. Bacterial and fungal communities in rhizosphere soil of wheat under MI differed significantly from those under WM and/or RI. This is partly supported by the results of Wang et al. (2012) who showed that cropping system exhibited only little impact on fungal and bacterial communities in rhizosphere soil of legumes and wheat. In another study, the composition of rhizosphere bacterial community was apparently altered by intercropping of maize and faba bean (Zhang et al., 2010), which is in contrast to our results. We speculate that the lack of a stronger effect of cropping regimes on bacterial and fungal communities in endosphere and rhizosphere observed in this study is attributed to the short growth period as the developmental stage of plants can influence microbial communities (Berg and Smalla, 2009; Zhang et al., 2011; Wearn et al., 2012; Robinson et al., 2016). This hypothesis is supported by a previous study on rhizosphere ammonia-oxidizing bacteria under different intercropping systems analyzed by DGGE (Song et al., 2007a). Here, intercropping showed a strong impact on these bacteria at anthesis but this effect was less pronounced at the seedling stage of the two crops.

Overall, we found different responses of fungal and bacterial communities toward cropping regimes (Table 6). The effects of cropping regimes were altered by crop species as well as plant compartment and differed between fungal and bacterial communities. We hypothesize that the contrasting effects of cropping regimes on microbial communities in soil and endosphere observed here and in other studies (e.g., Zhang et al., 2011; Wang et al., 2012; Yang et al., 2016) might be related to differences in soil type, plant species, and/or plant compartment investigated. It is well-known that these factors can influence microbial communities (Berg and Smalla, 2009; Wang et al., 2012; Wearn et al., 2012; Wemheuer et al., 2017). Moreover, plant species differ in their root exudates, which also can affect soil microbial communities (Berg and Smalla, 2009; Coleman-Derr et al., 2016). Thus, we further analyzed the impact of crop species and plant compartment on microbial communities (Table 5). Plant species significantly affected the composition of fungal communities but not of bacterial communities, explaining 3.1 and 1.7% of the variation, respectively. Plant compartment significantly altered bacterial and fungal community structure and explained 46.5 and 9.7% of the variance, respectively. The interaction of crop species and plant compartment explained 14.1 or 50.5% (faba bean) and 16.5 or 52.4% (wheat) of the variance in the dataset for fungal and bacterial communities, respectively. This indicates that fungal and bacterial communities respond differently to environmental changes. These results support the findings of Coleman-Derr et al. (2016) who analyzed fungal and bacterial communities of cultivated and native *Agave* species. Here, differences in fungal community structures were related to the biogeographical origin of the host species, while the structure of prokaryotic communities was primarily determined by the plant compartment.

**Table 6:** **Overview of results. Effect of cropping regime on bacterial and fungal richness, diversity, and community structure in faba bean and wheat**.

	**Bacteria**		**Fungi**	
	**Faba bean**	**Wheat**	**Faba bean**	**Wheat**
	**Cropping regime**		**Cropping regime**	
**BULK SOIL**				
Richness	^*^	**−**	**−**	**−**
Diversity	**+**	**+**	**+**	**−**
Structure	**+**	**+**	**+**	^*^
**RHIZOSPHERE**				
Richness	**−**	**+**	**−**	**−**
Diversity	**−**	**−**	**−**	**−**
Structure	**−**	**+**	**+**	**+**
**ROOTS**				
Richness	**+**	**−**	**−**	**−**
Diversity	^*^	**−**	**−**	**−**
Structure	**−**	**−**	**+**	**+**
**LEAVES**				
Richness	**−**	**−**	**+**	**−**
Diversity	**−**	**−**	**+**	**−**
Structure	**−**	^*^	**−**	^*^

+, Significant; −, no significant; ^*^, marginally significant.

We speculate that differences in plant physiology between *Fabaceae* and *Poaceae* including root topology or chemical composition (Roumet et al., 2008) are responsible for differences in endophyte communities. We further hypothesize that the different responses of fungal and bacterial endophytes toward cropping regimes, crop species, and plant compartment are related to different lifestyles of these microorganisms. According to Hardoim et al. (2008), there are three main categories of (bacterial) endophytes: obligate, facultative, and passive (passenger) endophytes. The latter colonize the plant as a result of stochastic events such as open wounds (Hardoim et al., 2008). It has been assumed that fungal endophytes remain restricted to a specific plant organ (Jaber and Vidal, 2010). Thus, many fungal endophytes in roots and shoots of several perennial forbs were tissue-specific (Wearn et al., 2012). However, some endophytic fungi are transmitted horizontally via soil- or air-borne spores (Sánchez Márquez et al., 2012), while other fungi are transmitted vertically, from parent to offspring via seeds (Hodgson et al., 2014). Another possible explanation is that plant species harbor a core set of seed-borne endophytes (Johnston-Monje and Raizada, 2011), which might also play a role in the present study. Overall, the results of the present study highlight that fungal as well as bacterial communities in different plant compartments should be analyzed in future studies.

### Abundant microbial taxa differ between cropping regime, plant species, and plant compartment

**Figure 7:**
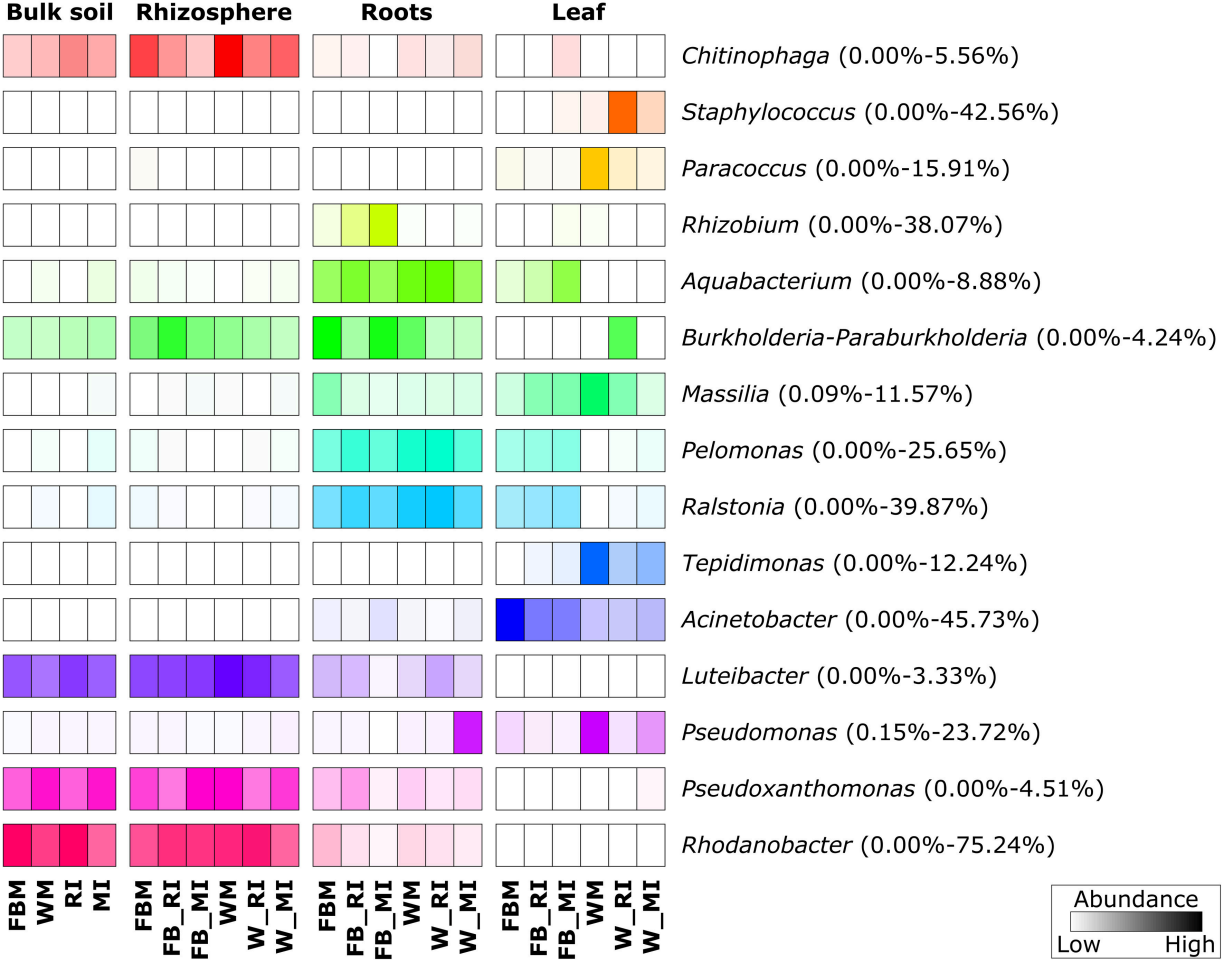
**Prominent bacterial genera in different plant compartments**. Only groups with an average abundance >1% are shown. The color code refers to sequence abundance, with high abundances (dark colored) and low abundances (light colored). Note that rows are standardized. FBM, all samples derived from faba bean monoculture; WM, all samples derived from wheat monoculture; FB_MI, faba bean samples from mixed intercropping; FB_RI, faba bean samples from row intercropping; W_MI, wheat samples from mixed intercropping; W_RI, wheat samples from row intercropping; MI, samples from mixed intercropping; RI, samples from row intercropping. Mean relative abundances of each taxa were calculated based on relative abundances calculated for each sample.

We further analyzed the abundances of the predominant fungal and bacterial taxonomic groups, as we found that fungal and bacterial communities respond in a crop species- and plant compartment-dependent manner to cropping regime. The most abundant bacterial genera showed clear trends regarding their preferred habitats (Figure 7). Several bacterial taxa such as *Ralstonia, Pseudomonas*, and *Massilia* were almost exclusively found in plant tissues while others including *Rhodanobacter, Luteibacter*, and *Chitinophaga* were mainly found in soil. The genera *Tepidimonas, Acinetobacter, Paracoccus*, and *Staphylococcus* were almost exclusively detected in leaves whereas *Rhizobium* was more abundant in roots. The abundances of predominant bacterial genera differed not only between the four plant compartments, but also between cropping regimes and crop species. Some genera including *Paracoccus* and *Tepidimonas* were mainly found in wheat leaves with the highest abundance in monocultured wheat plants.

**Figure 8:**
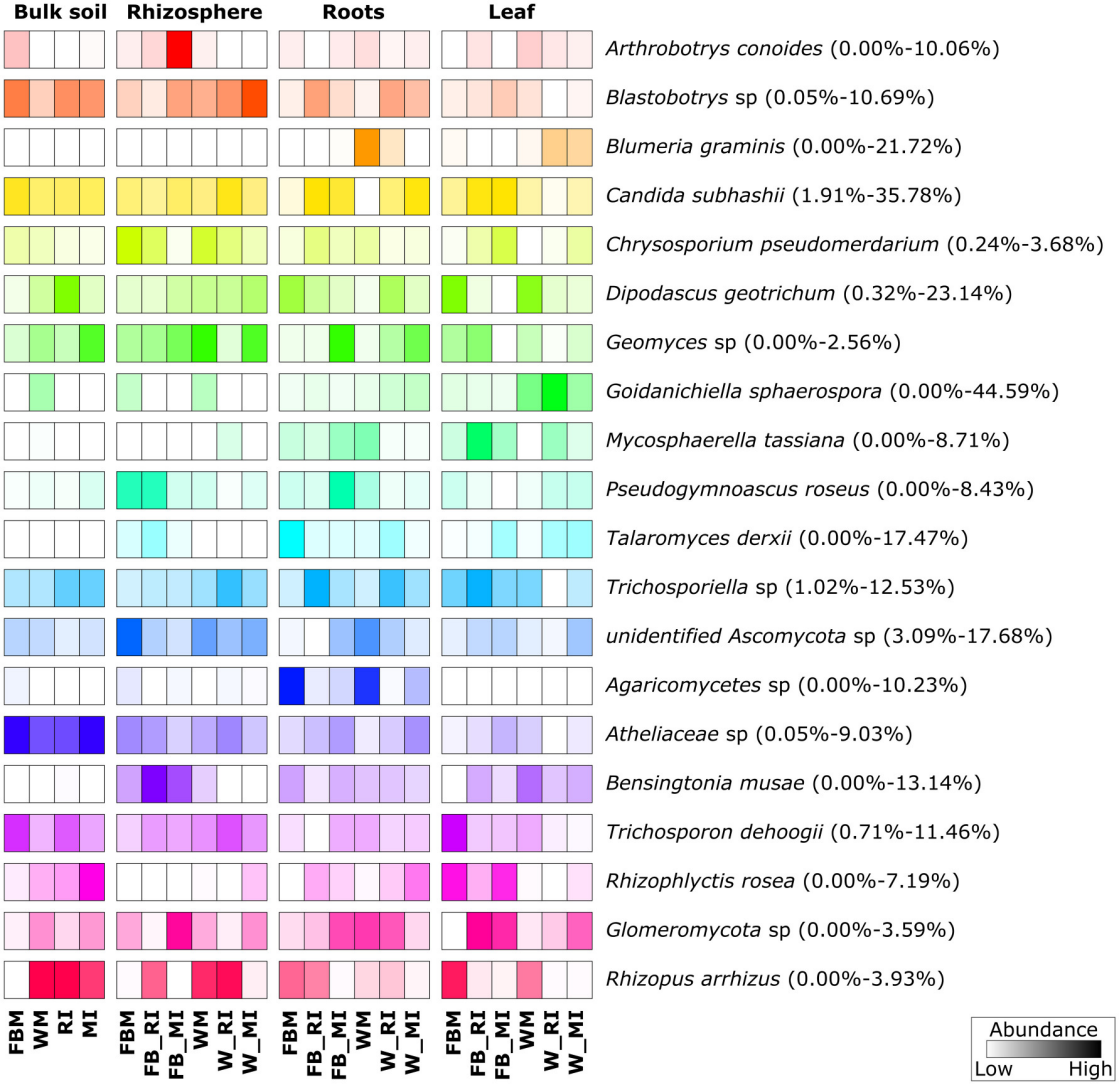
**Prominent fungal species in different plant**. Only species with an average abundance >1% are shown. The color code refers to sequence abundance, with high abundances (dark colored) and low abundances (light colored). Note that rows are standardized. FBM, all samples derived from faba bean monoculture; WM, all samples derived from wheat monoculture; FB_MI, faba bean samples from mixed intercropping; FB_RI, faba bean samples from row intercropping; W_MI, wheat samples from mixed intercropping; W_RI, wheat samples from row intercropping; MI, samples from mixed intercropping; RI, samples from row intercropping. Mean relative abundances of each taxa were calculated based on relative abundances calculated for each sample.

For fungi, we did not observe such clear patterns (Figure 8). However, some fungal species such as *B. graminis* (causative agent of powdery mildew) and *Mycosphaerella tassiana* were mainly found in plant tissues, whereas others including *D. geotrichum, Geomyces* sp., *C. subhasii*, or *T. dehoogii* were detected in almost all plant compartments and both crop species regardless of the cropping regime. *Goidanichiella sphaerospora* was predominant in wheat leaves while an uncultured member of the *Agaricomycetes* was mainly found in roots of monocultured crop plants. *Talaromyces derxii* was found in high abundances in roots of monocultured faba beans.

Statistical analysis revealed that several of the abundant bacterial genera and fungal species were significantly affected by cropping regime, crop species, and/or plant compartment (Tables S8, S9). For example, the abundances of *Rhizobium* in rhizosphere soil samples differed significantly between intercropped faba bean plants and wheat plants under mono- as well as intercropping. The abundances of *Paracoccus* differed significantly between root endosphere samples of faba bean under RI and FBM, leaf endosphere samples of monocropped faba bean and faba bean under MI, monocropped and intercropped wheat. Moreover, rhizosphere samples of faba bean under RI and FBM as well as bulk soil samples of monocropped faba bean and wheat plants and plants under intercropping showed significant changes in the abundances of this genus. In addition, abundances of *M. tassiana* and *B. graminis* in leaf endosphere were significantly affected by crop species as well as cropping regime. The abundances of *Goidanichiella sphaerospora* differed significantly between leaf and root endosphere samples of faba bean under the different cropping regimes (FBM, RI, MI) and leaf endosphere samples of wheat under the cropping regimes WM and MI.

We identified several bacterial and fungal taxa with plant growth-promoting potential, such as *Burkholderia, Pseudomona*s, *Rhizobium, C. subhashii*, and *Streptomyces bungoensis*. For example, the fungal species *T. derxii* can produce several secondary metabolites with antibacterial activity (Zhai et al., 2016). Members of the genera *Burkholderia, Pseudomonas*, and *Rhizobium* are well-known as plant growth-promoting bacteria and/or for the production of secondary metabolites including antibiotics or antifungal compounds (Lodewyckx et al., 2002; Lugtenberg and Kamilova, 2009). Moreover, several isolates of *Rhodanobacter* (Kostka et al., 2012) and *Massilia* (Zhang et al., 2006) were able to reduce nitrate indicating that these genera play a key role in the nitrogen cycle. Interestingly, *Rhizobium* was mainly found in roots of intercropped faba bean, with the highest abundance in MI. Legumes such as faba bean are well-known for their symbiosis with nitrogen-fixing rhizobia including members of the genus *Rhizobium* (Lugtenberg and Kamilova, 2009). We speculate that the higher abundance of this genus in intercropping regimes is related to a higher selection of faba bean for these bacteria as intercropped faba bean and wheat plants compete for nitrogen (Zhang and Li, 2003; Mariotti et al., 2009).

In addition to beneficial microorganisms, we detected various fungal phytopathogens, such as *B. graminis* and *Rhizopus arrhizus*, as well as bacterial genera containing widely recognized human and plant pathogens, i.e., *Ralstonia* and *Staphylococcus*. However, obtained sequences of *Ralstonia* and *Staphylococcus* were predominantly affiliated to uncultured bacteria within these genera. In a previous study on the impact of pest management on bacterial endophyte communities in two grapevine cultivars, *Ralstonia* was the dominant genus in these communities (Campisano et al., 2014). Recently, members of the genus *Ralstonia* were observed as endophytes in several grass species (Wemheuer et al., 2017). The observation of these fungi and bacteria in healthy plants indicates that plant endosphere and rhizosphere are an important reservoir for several potential plant as well as human and/or animal pathogens (Mendes et al., 2013; Hardoim et al., 2015). As consequence, a better understanding of the plant microbiome and its responses to cropping regimes is needed.

### Bacterial and fungal taxa associated with cropping regimes, crop species, and plant compartment

As we found different responses of abundant fungal and bacterial taxa to cropping regimes, we performed a multipattern analysis to investigate which microorganism are significantly associated with those regimes (Table S10). In general, soil communities harbored more associated OTUs than endophyte communities, most probably related to higher sequence numbers in soil compared to endosphere samples. The highest number of associated fungal and bacterial OTUs was observed for rhizosphere soil of wheat plants. In general, more endophytic bacteria were associated with faba bean than wheat while the opposite was detected for fungal OTUs. Interestingly, only one OTU belonging to *Staphylococcus* was significantly associated with leaves of wheat under RI.

We identified some species associated with rhizosphere and bulk soil of wheat and/or faba bean, such as *Sphingomonas* sp. C0503 and an uncultured fungal member of *Conlarium* sp. Other microorganisms including *C. subhasii, Massilia*, or *Rhodanbacte*r were significantly associated with endosphere and soil compartments. The yeast *C. subhashii* might play an important role in plant growth promotion as this yeast strongly antagonized a wide range of filamentous fungi (Hilber-Bodmer et al., 2017). The predominant fungus *D. geotrichum* was associated with the root endosphere of wheat under RI. In addition, this fungus was significantly associated with the leaf endosphere of monocultured faba bean as well as the bulk soil of faba bean under RI.

Other fungi and bacteria were only associated with one plant compartment and/or one crop species. For example, the antibiotic-producing *S. bungoensis* (Eguchi et al., 1993) was significantly associated with roots of monocultured faba bean. *Moraxella osloensis* were only associated with leaves of faba bean under MI, while *Chrysosporium pseudomerdarium* was significantly associated with faba bean rhizosphere in monoculture and intercropping regime RI. The last-mentioned fungus can produce gibberellins and thus might promote the growth of plants (Hamayun et al., 2009). The association of the bacterium *M. osloensis* is interesting due to its potential for the biological control of slugs (Tan and Grewal, 2001). The nematode-trapping fungus *A. conoides* (Yang et al., 2007) and an uncultured member of the *Glomeromycota* were significantly associated with the rhizosphere of faba bean under cropping regime MI. The *Glomeromycota* encompass the arbuscular mycorrhizal fungi which are often associated with crops such as wheat and barley (Jensen and Jakobsen, 1980). Recently, it has been shown that mycorrhizal colonization in wheat/faba bean intercropping systems stimulates the transfer of fixed N from faba bean to wheat (Wahbi et al., 2016) and thus can promote plant growth. Although we identified several associated fungi and bacteria with plant growth-promoting potential, further research is needed to understand the role of these microorganisms in the plant microbiome and their response to cropping regimes.

### Bacterial and fungal co-occurrence in the plant compartments

**Figure 9:**
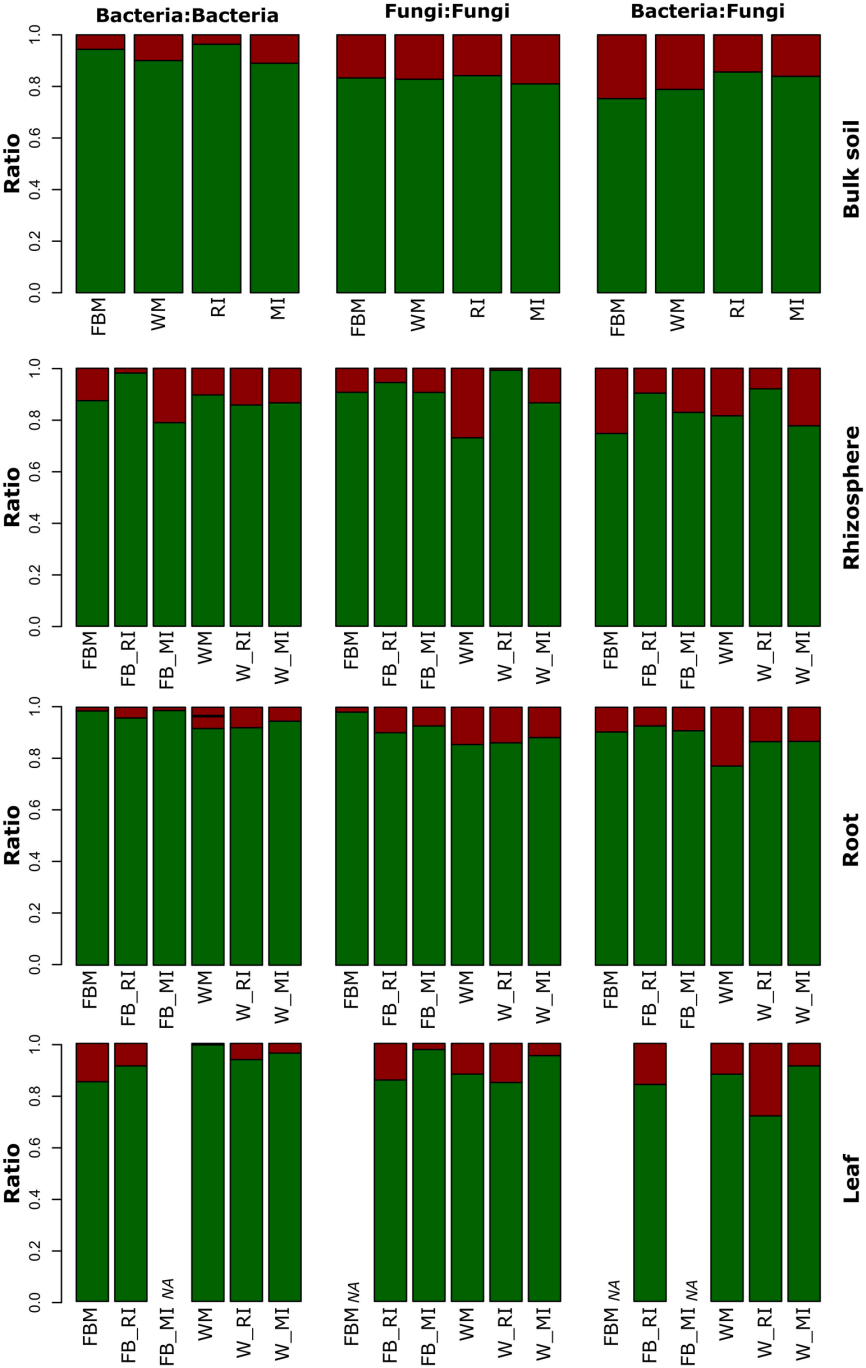
**Positive and negative relative interactions in different plant compartments of faba bean and wheat with regard to cropping regime**. Positive and negative relative interactions are shown in green and red, respectively. Bulk soil samples of intercropping regimes consisted of both crop plants. FBM, all samples derived from faba bean monoculture; WM, all samples derived from wheat monoculture; FB_MI, faba bean samples from mixed intercropping; FB_RI, faba bean samples from row intercropping; W_MI, wheat samples from mixed intercropping; W_RI, wheat samples from row intercropping; MI, samples from mixed intercropping; RI, samples from row intercropping; NA: data not available due to low sample numbers.

To investigate the effect of cropping regimes on inter- and intra-domain interactions of fungi and bacteria, we calculated the number of significant correlations between OTUs for each compartment (Figure 9, Table S11). Positive interactions (indicating species co-occurrence) are regarded indicative for cooperation, whereas negative interactions indicate avoidance or competition. The abundances of intra-domain negative interactions of bacteria increased strongly in the rhizosphere of faba bean under MI compared to RI and FBM while negative fungal intra-domain interactions remained stable (Figure 9). In contrast, negative fungal intra-domain interactions increased in wheat rhizosphere under MI compared to RI and WM. The number of negative inter-domain correlations between fungi and bacteria decreased in bulk soil of faba bean and wheat under RI and MI compared to monocultures which might be attributed to beneficial effects. Moreover, we observed plant species-dependent differences. The number of negative interactions for bacteria in wheat roots was higher compared to those in faba bean roots. As mentioned above, legumes and grass species differ in their physiology (Roumet et al., 2008), which might also affect interactions within the plant microbiome. Another possible explanation is that intra- and interspecific competition between intercropped plants had different effects on the plant species and thus on their associated microbial communities.

Only a few intra-domain fungal interactions were detected in root and leaf endosphere as well as rhizosphere of faba bean and/or wheat (Figure 9, Table S11). These findings are in contrast with a recent study of Yan et al. (2015) who observed a strong intra-phylum competition between endophytic fungi and differing occupation of niches within the leaves. Negative associations between co-occurrence of fungal endophyte species were also found for *Cirsium arvense* which might be related to the order of endophyte colonization (Gange et al., 2007). In another study, negative correlations were observed between mycorrhizal colonization and endophyte presence in roots of the two herbaceous grassland plant species *C. arvense* and *Plantago lanceolata* (Wearn et al., 2012). We speculate that the contrasting findings are explained by differences in the growth stage of the plant species investigated as we investigated young plants. As mentioned above, some endophytic fungi are vertically transmitted in plants (Hodgson et al., 2014). This provides advantages for fungal endophytes as the order of colonization of endophytes within leaves can be critical for the determination of the community structure (Gange et al., 2007). The authors hypothesized that when endophytes are growing within the host plant, then certain fungal species may suppress or exclude other fungi resulting in negative relations between these species. Thus, we hypothesize that the negative interactions for fungi become clearer when older plants are investigated. Nonetheless, the results of the above-mentioned studies and our study highlight that it is essential to understand not only interactions between plant host, its microbiome and their environment, but also the diverse interactions within the plant microbiome.

### Study limitations and future experiments

Several technical and biological limitations of this study must be considered. First, we cannot exclude the possibility that we sampled epiphytic root or phyllosphere microbes when analyzing endophyte communities. We verified the effectiveness of the surface sterilization protocol by two different methods (see Materials and Methods) indicating that the protocol used is sufficient for the surface sterilization of plant tissues. Another caveat of this work and other studies on plant-associated microbial communities (Desgarennes et al., 2014; Coleman-Derr et al., 2016) is the use of different DNA extraction kits depending on the sample type. This can be explained by the fact that the DNA kits are not equally suited for different starting materials and the respective contaminations. As consequence, direct comparison of endophyte and soil communities should be treated with caution when using different DNA extraction kits.

Another limitation of this study is the problem of primer bias in PCR (Anderson and Cairney, 2004; Hong et al., 2009). Hong et al. (2009) suggested that any particular primer pair enables theoretical recovery only up to 50% of the bacterial diversity in a given sample. To encourage amplification of all bacterial and fungal groups, we tested different primer combinations before conducting the experiment, e.g., the primers of the second PCR directly without the first PCR. This gave us more than 90% plant-derived contaminations in both data sets. We also tested universal primers, e.g., the primers recommended by Klindworth et al. (2013) or Toju et al. (2012) for the V3 and V4 region of the bacterial 16S rRNA gene or for the fungal ITS region, respectively. We had a similar high contamination rate of more than 99% plant-derived sequences. Nonetheless, most sequences were removed in the initial processing steps as only high-quality, double-chimera checked sequences should be used in the analysis. Moreover, we performed nested PCR with three technical replicates for each sample in the second PCR. In total, five biological replicates per treatment were analyzed (see Materials and Methods).

Another technical limitation of this study was that we assessed fungal and bacterial communities associated with relatively young plants (~30 days old after germination). Previous studies showed that the plant developmental stage had a strong effect on plant microbiome (Berg and Smalla, 2009; Wearn et al., 2012; Robinson et al., 2016). In contrast, plant developmental stages had only a minor impact on community assemblage for both bacterial and fungal community assemblages of sugarcane microbiome (de Souza et al., 2016). Nevertheless, it would be interesting to analyze different plant developmental stages and validate how this affects the response of microbial communities toward cropping regimes. We speculate that effects of cropping regimes on microbial diversity and community structures will become more evident after a longer growth period.

A major limitation of the study presents the low sequencing coverage, which permitted the description of fungal and bacterial communities at 20 and 276 sequences, respectively. We chose these minimal sequence thresholds to balance sufficient replicate numbers per sample groups and to assure minimal reliability of the microbial profiles. Certainly, enhanced sequence coverage would permit more in-depth and solid analyses. Another caveat of this study is that we detected unusually low OTU numbers, especially in bulk soil samples, compared to OTU numbers in other studies (e.g., Coleman-Derr et al., 2016; Hartman et al., 2017). It seems possible that the potting soil, the greenhouse conditions, and/or the utilized profiling approach are responsible for the low OTU numbers observed in the present study. However, a similar low fungal diversity in field soils was observed by Kazeeroni and Al-Sadi (2016). Commercial potting soils are often steamed to reduce the occurrence of pathogens. This treatment lowers the microbial load and could result in lower OTU diversity. In their review on the influence of experimental conditions on the structure and diversity of the plant microbiome, Berg et al. (2015) concluded that the microbial diversity is strongly influenced by the environmental settings, as exemplified by agricultural systems or plants raised in pot experiments. The low microbial diversity in the endosphere observed in our study might be related to the short growing period of the plants. Another explanation is that the cultivation of plants led to lower levels of prokaryotic diversity compared with native plants, which was observed in two recent studies investigating the plant microbiome of different *Agave* species (Coleman-Derr et al., 2016) and the rhizosphere microbiome of *Beta vulgaris* spp. *maritima* and modern sugar beets (Zachow et al., 2014). Finally, the 2-step nested approach with 70 cycles of PCR might disproportionally amplify abundant taxa resulting in community profiles of low diversity. Future experiments are required to validate these possible explanations.

The last technical limitation is that we performed a greenhouse pot experiment using commercial plant substrate. Nonetheless, greenhouse experiments allow controlled conditions with a simplification of environmental heterogeneity. In addition, the results of potting mix experiments are easier to interpret as the results are often too complex when natural field soils are used (Ofek et al., 2009). However, a previous study showed that soil type (natural vs. potting soil) had a strong influence on the rhizosphere and endosphere microbiome of sugar beet (Zachow et al., 2014). According to Berg and Smalla (2009), it is difficult to extrapolate results of climate chamber or greenhouse experiments to natural field conditions if the natural rhizosphere cannot develop due to the experimental design. As non-sterilized soil substrate can provide plants with a microbiome (Berg et al., 2015), we did not sterilize the soil substrate prior to usage.

## Conclusion

The present study provides first insights into fungal and bacterial co-occurrence patterns in different plant compartments of two important crop plant species. Our major results were that plant compartment and plant species altered the effects of cropping regimes on microbial communities as well as on microbial interactions. Moreover, we observed different responses of fungal and bacterial communities toward cropping regimes. These findings suggest that future studies should concentrate not only on bacterial and/or fungal communities in one plant compartment and/or one plant species. Although the results of greenhouse experiments cannot be transferred to field conditions, they can serve as background for further field studies. Consequently, the next steps would be to investigate microbial communities in different plant compartments of faba bean and wheat grown as mixture and in monoculture under field conditions.

## Author contributions

SV and FW conceived and guided the research. SG, KK, BW, BP, and FW were involved in data acquisition and analysis. SG, KK, BW, BP, and FW wrote the first draft of the manuscript. All authors contributed to interpretation of results and were involved in critical revision and approval of the final version.

### Conflict of interest statement

The authors declare that the research was conducted in the absence of any commercial or financial relationships that could be construed as a potential conflict of interest.
